# Covid-19 pandemic by the “real-time” monitoring: the Tunisian case and lessons for global epidemics in the context of 3PM strategies

**DOI:** 10.1007/s13167-020-00207-0

**Published:** 2020-04-25

**Authors:** Lotfi Chaari, Olga Golubnitschaja

**Affiliations:** 1 University of Toulouse, IRIT - INP-ENSEEIHT (UMR 5505), 2 rue Charles Camichel, BP 7122 Toulouse Cedex 7, France; 2 Predictive, Preventive and Personalised (3P) Medicine, Department of Radiation Oncology, Friedrich-Wilhelms-University Bonn, Bonn, Germany

**Keywords:** Covid-19, SARS-CoV-2, Infection, Pandemic, Epidemics, Predictive preventive personalised (3P) medicine, Bottle-neck, Strategy, Targeted protective measures, Multi-professional expertise, Laboratory medicine, Policymaking, Salami-tactic, Population screening, “Real-time” monitoring, Home isolation, Comorbidities, Related mortality, Depression, Suppressed immune defence, Suicide, Psychotic attitude, Violence, Anti-gene, Anti-body, Test, Individual outcomes, Ethics, Titanic, Triage, Economy

## Abstract

Covid-19 is neither the first nor the last viral epidemic which societies around the world are, were and will be affected by. Which lessons should be taken from the current pandemic situation? The Covid-19 disease is still not well characterised, and many research teams all over the world are working on prediction of the epidemic scenario, protective measures to populations and sub-populations, therapeutic and vaccination issues, amongst others. Contextually, countries with currently low numbers of Covid-19-infected individuals such as Tunisia are intended to take lessons from those countries which already reached the exponential phase of the infection distribution as well as from those which have the exponential phase behind them and record a minor number of new cases such as China. To this end, in Tunisia, the pandemic wave has started with a significant delay compared with Europe, the main economic partner of the country. In this paper, we do analyse the current pandemic situation in this country by studying the infection evolution and considering potential protective strategies to prevent a pandemic scenario. The model is predictive based on a large number of undetected Covid-19 cases that is particularly true for some country regions such as Sfax. Infection distribution and mortality rate analysis demonstrate a highly heterogeneous picture over the country. Qualitative and quantitative comparative analysis leads to a conclusion that the reliable “real-time” monitoring based on the randomised laboratory tests is the optimal predictive strategy to create the most effective evidence-based preventive measures. In contrast, lack of tests may lead to incorrect political decisions causing either unnecessary over-protection of the population that is risky for a long-term economic recession, or under-protection of the population leading to a post-containment pandemic rebound. Recommendations are provided in the context of advanced predictive, preventive and personalised (3P) medical approach.

## Introduction

The current Coronavirus pandemic [1] is the most dramatic healthcare crisis linked to acute and highly infectious disease never faced by the global world since begin of the twentieth century. The Coronavirus infectious disease has been first diagnosed in China. To restrict the epidemic spread was extremely challenging for China, since no prior information was available about the new virus—the circumstance which caused the “latent” observation period prior to decide on containment as a main and strict measure [2]. All other countries involved are facing this pandemic threat with more or less delay compared to each other: after China, the pandemic wave appeared first in Asia followed by Europe deeply touching Italy, France and Spain. The spread to America, Australia and Africa then followed.

Since the pandemic spread in China, a series of studies have been initiated in parallel for therapeutic [3, 4] and vaccination [5] purposes. However, due to the current deficit in the targeted treatment of Covid-19, protective measures present the main strategy stream that most countries apply mainly at the national and regional levels. The main goal of all the protective measures is to slow down the virus propagation in order to adapt the number of severely sick people to the limited capacity of corresponding health system providing an adequate care, and therefore to keep the morbidity level at a minimum. However, to make preventive strategies working, they should rely on the case-adapted multi-parametric predictive models which are still missing for the Covid-19 pandemic, due to many unclear parameters such as the real number of infected persons in the country and specific characteristics of individuals particularly predisposed to severe disease forms. Consequently, protective recommendations are highly generalised being limited to the social distancing [6] and hygiene rules usual for any flu episode.

In addition to generalised protection, the diagnostic approach is one of the main recommendations provided by the World Health Organization (WHO). To this end, the most reliable way to detect the infection is to perform the anti-gene-targeted PCR analysis [7], in order to assess the presence of Covid-19 RNA (ribonucleic acid) in the hosting individual. However, this approach demands the “real-time” monitoring, since the negatively tested individual today, tomorrow may easily turn out as positively tested. Therefore, more stable individual results and population statistics would be provided by the anti-body tests to detect everyone who diseased on Covid-19.

Because of low testing capacity especially at the very beginning of the pandemic spread, most countries performed tests generally to persons suffering from unclear and advanced symptoms, or individuals who evidently have been in contact with infected ones.

Countries facing the follow-up infection distribution being luckily delayed in pandemic spread such as Tunisia, certainly have to make a good use of currently accumulated knowledge. The main objective of this paper is to support and to direct the learning process. Specifically in Tunisia, per evidence the infection has been imported primarily from Italy and France. Since the very beginning of the pandemic spread in the country, Tunisian authorities intended to adopt a containment strategy in order to limit the virus propagation, the main barrier for which is a low testing capacity. Further, there is a significant discrepancy in tests performance amongst the regions within the country. How to proceed in this particular condition and what are the main lessons from Covid-19 pandemic for the moment?

The current article is not ambitioned to provide a completed analysis of the Covid-19 pandemic that will be possible only when all the aspects will be clarified that currently is definitely not the case. However, some fragmental information available provides a good platform for preliminary analysis in the global context. Based on that, expert recommendations for comprehensive 3PM strategies are presented below.

### Analytical focus

The analysis was performed to estimate Covid-19-related mortality risks. This evaluation utilised regularly published statistics, comparative studies performed in Italy and France [8] and data reported by the Ministry of Health in Tunisia.

### Comparative analysis of the infection evolution

**Fig. 1:**
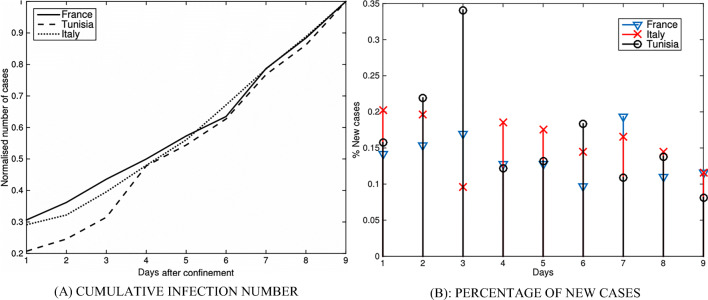
Evolution **A** of infection numbers and **B** of percentage of new cases

Here the evolution of the infection propagation is presented as compared between France, Italy and Tunisia—the countries which established containment instructions with some delay against each other depending on the local pandemic expansion. Figure 1 displays the evolution of infection in three countries of comparison for 9 days after the corresponding containment date.

Figure 1A displays the normalised (with respect to the total number of inhabitants per country) curves of cumulative infected cases for the same period of time, while Fig. 1B displays the new cases as percentage from the total numbers of infected persons. To this end, for the data collection comprises for both—France and Italy the period of time from February 15th to March 30th. For Tunisia, where the pandemic started with some delay, the recorded period is from March 2nd to 29th.

From the images presented in Fig. 1, it is evident that the pandemic evolution is rather similar for all three countries; in particular, they are conjoint when the recorded numbers are reaching their maximum. Similarly for all three counties, the percentage of new cases starts to get stabilised after 5 containment days. Summarising, the countries of comparison have applied similar containment rules and share similar evolution. However, compared to France and Italy, Tunisia started the containment almost 2 weeks earlier considering the level of pandemic spread. This preventive measure allowed Tunisia so far to reduce the absolute number of infected people. Consequently, Tunisia currently counts less than 400 identified infection cases, while France and Italy count more than 45,000 and 101,000 cases, respectively. Table 1 presents the identified infection numbers in all three countries, as well as their respective containment dates.

**Table 1:** Pandemic reflection in France, Italy and Tunisia; the early containment strategy allowed for reducing the absolute number of detected cases in Tunisia

	France	Italy	Tunisia
Containment date	March 17th	March 9th	March 22nd
Absolute number of cases recorded before the containment	7730	9172	89
Absolute number of cases recorded till March 30th	44,550	101,739	394
Population in millions	67	60	11
% infection cases per thousand of inhabitants	0.66	1.69	0.035

From Table 1, it is evident that the percentage of infection cases per thousand of inhabitants was significantly lower in Tunisia compared to both - France and Italy.

To summarise, the pandemic evolution in Tunisia is similar to this in Italy and France. However, the early containment strategy allowed for reducing the absolute number of detected cases as well as the relative number of cases recalculated through the entire population.

**Fig. 2:**
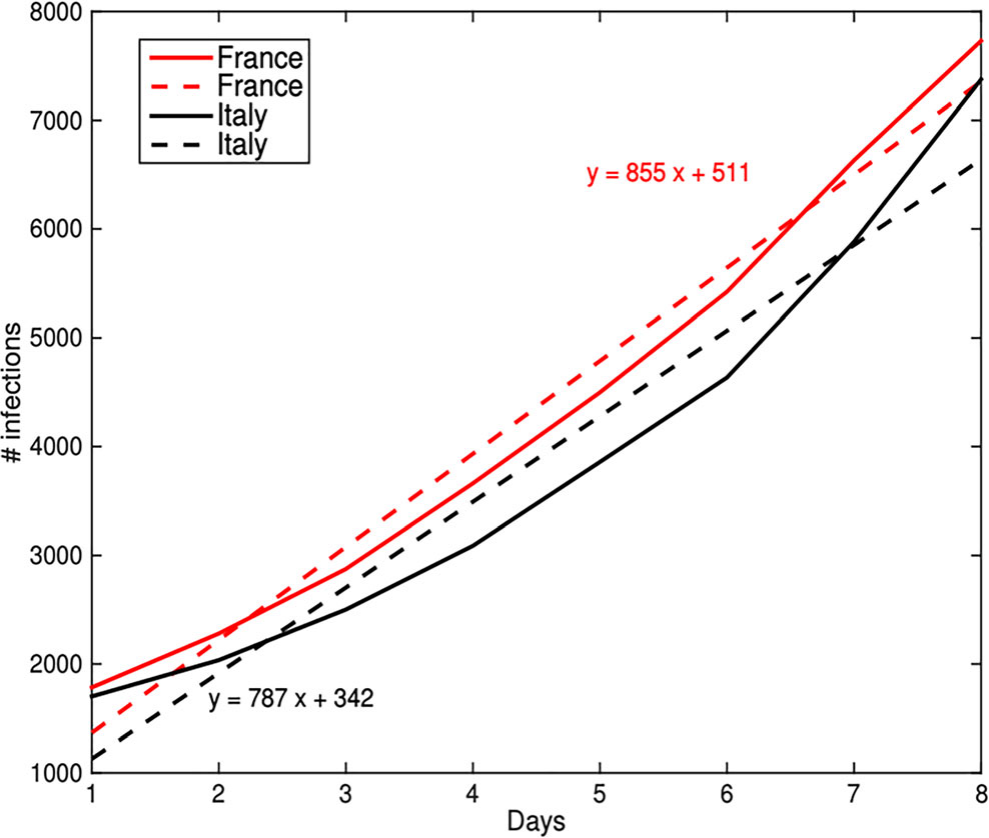
Evolution of the infection numbers a week before containment (solid lines) and their linear approximation over the period (dashed lines)

To go beyond this conclusion, we have estimated the number of infected cases which Tunisia could deal with, if the containment would be delayed by a week. Doing this, we have used estimations based on the data recorded for France and Italy assuming that one week before containment the evolution curve is a short-term linear one and follows the simple equation

1 $$ y= ax+b $$

where *x* is the day and *y* stands for the cumulative infection number. The parameters *a* and *b* have to be estimated using a least squares (LS) estimation for example. Contextually, Fig. 2 illustrates the evolution of the infected cases for France and Italy

corresponding to the respective weeks before containment. The figure also displays the estimated linear curves utilising the LS method.

Although the evolutions of comparison are very similar to each other, the evolution speed for France is slightly higher despite the lower number of infections. Further, by assuming that the evolution for Tunisia would follow the mean behaviour based on the two estimated curves, if the containment would have been delayed by only weeks, the corresponding number of infection cases would be equal to 5.836 on March 29th, while on this day the officially recorded number was 362. In the next section, the analysis is presented to understand, whether this remarkable difference is due to early containment effect only.

### The protective strategy in Tunisia: pros and cons

Here, we do analyse a currently applied protective strategy in Tunisia resulting in relatively low numbers of officially recorded Covid-19 infection cases. To be noted, the laboratory testing capacity (PCR) in Tunisia is highly limited compared with France and Italy. Indeed, the maximum recorded tests made in Tunisia are 724 daily, while only the city of Marseille in France provides than 11,000 tests per day. To this end, currently Tunisia is intensively working on increasing the testing capacity significantly, specifically focusing on PCR anti-gene tests. Further, Tunisia follows the same targeted test strategy as practised in France and Italy: the tests are done principally for individuals who evidently were in contact with Covid-19-infected persons or patients presenting severe symptoms of manifested disease. Taking these facts into account, it is getting clear that the real numbers of infected cases should be much higher than the officially recorded ones. This conclusion is well supported by the study performed by the University of Göttingen in Germany, which demonstrates that on average only 6% of actual SARS-CoV-2 infections are detected worldwide, and the real number of infections may have reached several tens of millions in April 2020 [9]. Furthermore, the tests performed in Tunisia till now do not provide representative statistics for the whole country, since the density for both—the population and tests performance—differ significantly from region to region in the country. Contextually, alarmingly high death rates in some regions such as Sfax have been recorded. To this end, Sfax is the city with the second highest population density hosting almost 9% of the entire population in the country. In this city, fourteen Covid-19 cases were officially registered on March 29th with 3 lethal outcomes that means 21% mortality. In contrast, in Sousse the mortality rates of 4% are recorded. At the same time, the so-called big Tunis region (about 25% of the entire population) has recorded one lethal outcome (0.66%) within 150 officially recorded cases. Specifically in this region, the anti-gene PCR tests are most effectively performed utilising the lab medical capacity of two specialised centres from altogether four currently existing in Tunisia (status quo on March 30th, 2020) which conforms with the recommendation of WHO [10] to broadly apply testing strategy that helps to decrease the overall mortality. Indeed, patients identified at early stages of the disease could benefit from adapted treatments. In contrast, for the moment the Sfax region does not host any specialised medical centre with a consequence of extremely low testing activity in the region. The majority of infected individuals have not been tested. As a matter of fact, two of three deaths correspond to persons who have not been provided a medical care; they died at home and postmortem have been positively tested for the Coronavirus infection as the cause of their death.

## Conclusions

Per evidence, deficits in the testing procedure led to increased mortality due to undetected infection cases. In the context of Tunisia, the main problem is the testing inequality amongst the regions: the testing is focused on the capital but is not adequately performed through over all the regions. Further, highly limited testing capacity in the country causes a huge discrepancy between the officially recorded and real infection cases. Indeed, if according to the already known statistics, the mortality rates of the Sfax region get extrapolated for the entire country, the current real number of infected individuals would be as high as 5000 cases for Tunisia now. This estimation is indeed close to the numbers resulted from the above presented model (see the above presented “Comparative analysis of the infection evolution”). The official statistics revealed only 10% of tested people were Covid-19 positive. Taking this number in consideration, more than 45,000 tests have to be performed in order to limit the virus propagation before stopping containment. However, the tests have been performed only for persons with severe disease symptoms and after evident contact to infected patients. Therefore, assuming that the real number of infected persons is four to ten times higher, at least 150,000 tests have to be performed prior to decide on stopping containment. Further, the “real-time” anti-gene monitoring is essential, since persons negatively tested today can be positively tested tomorrow. To this end, anti-body tests would provide more stable data on how many persons have been diseased. Finally, a relatively cheap but reliable X-ray diagnostic approach can also be considered utilising novel artificial intelligence tools [11].

## Lessons from the Covid-19 pandemic: a global approach in the context of 3P medicine

Covid-19 is neither the first nor the last viral epidemic which societies around the world are, were and will be affected by. Which lessons should be taken from the current pandemic situation?

- More prediction is essential to avoid mistakes in the decision making process.
- Retrospective analysis of data is needed to create a valid scenario of current and coming epidemics. Thereby, evidence-based monitoring, providing reliable data and international data exchange, is crucial.
- Creation of prototype algorithms as the basic predictive tool to be prepared for the next epidemics is highly desirable.
- Laboratory medicine is central in combating pandemics. An excellent example has been provided by Iceland with randomised tests to the population in order to estimate the real number of infected persons. To this end, the “real-time” monitoring is essential utilising both the anti-gene and anti-body tests.
- Multi-professional expertise is an obligation to create effective strategies including laboratory medicine, epidemiology, virology, immunology, microbiology, pulmonology, psychology, psychiatry, disease-modelling and bioinformatics, amongst others.
- Resolute targeted prevention instead of “salami” tactic should be considered by policymakers. Delayed and unconsolidated region-dependent measures as well as highly heterogeneous attitude ranging from “doing nothing”, “observing what other are doing” up to within few days changing mind from “no matter” to “facing a severe problem” is misleading for populations which under the epidemic condition need particularly clear statements and well justified strategies.
- Personalisation of protective measures and treatments are essential by evidence-based identifying groups at risk and stratifying patients based on the individualised profiling.
- Ethical aspects: under pandemic conditions, the triage presents a “Titanic”-unlike dilemma, namely which groups of individuals should be prioritised for which kind of medical services. In case of Titanic, the decision was relatively simple: children and women have been prioritised for placing into the limited number of boats. More complicated is the “triage” for infected individuals under epidemic conditions, due to highly complex situations. Under the optimal scenario, a “bottle-neck” should be minimised and targeted protection should be provided to any group in the population. Under the current Covid-19 pandemic, these include timely epidemiologic data collection, sufficient laboratory tests, well in time organised isolation of elderly, protective measures to employees at points with high density of contacts (shops, schools, transport, etc.), financial support to low-income groups and economically weak enterprises. From the ethical point of view, again, predictive algorithms are needed to create the most effective targeted preventive measures and personalised care to citizens by implementing 3PM concepts.
- Relevance of economic aspects for individual health outcomes: The current pandemic caused unemployment and economic depression at individual and population levels frequently leading to a cascade of comorbidities including depression, suppressed immune defence, suicide, psychotic attitude and violence, amongst others, resulting in related mortality. Therefore, a well-justified balance between ordered limitations to the population (home isolation, temporary unemployment etc.) aiming at decreasing the number of new Covid-19 infected cases on the one hand and well-controlled timely normalisation of the societal functioning on the other hand is essential based on reliable predictive algorithms.

The above provided recommendations are conform with the 3PM-related guidelines published by the European Association for Predictive, Preventive and Personalised Medicine [12].
